# WEIGHT LOSS COMPARISON AFTER SLEEVE AND ROUX-EN-Y GASTRIC BYPASS: SYSTEMATIC REVIEW

**DOI:** 10.1590/0102-672020190001e1474

**Published:** 2019-12-20

**Authors:** Fernando de BARROS, Mayara Galisse NEGRÃO, Giovana Galisse NEGRÃO

**Affiliations:** 1 Department of General and Specialized Surgery, Faculty of Medicine, Fluminense Federal University, Niterói, RJ, Brazil; 2 Medical College, University of Franca, Franca, SP, Brazil

**Keywords:** Weight loss, Obesity, Bariatric surgery, Gastric bypass, Sleeve, Perda de peso, Obesidade, Cirurgia bariátrica, Bypass, Sleeve

## Abstract

**Introduction:**

Bariatric surgery is currently the gold standard treatment for obesity. The two most accomplished surgeries are the Roux-en-Y gastric bypass and the sleeve gastrectomy, and controversies exist in which is better.

**Objective:**

To compare the two techniques in relation to weight loss with at least five years of follow-up.

**Methods:**

Search in Medline, PubMed, Embase, SciElo, Lilacs, Cochrane databases from 2001 (beginning of vertical gastrectomy) until 2018, using the following headings: “sleeve” or “sleeve gastrectomy” combined with “gastric bypass” or “Roux-en-Y gastric bypass”, “weight loss” and “clinical trial”. Criteria for inclusion of articles were patients aged between 18 and 65 years; clinical trial; comparison between the two techniques; minimum five-year follow-up; outcome with weight loss assessment.

**Results:**

The initial search identified 1940 articles, of which 185 publications were identified as clinical trials. One hundred and forty-one were excluded, 67 because they did not compare the two techniques, 57 not addressed weight loss and 17 were repeated articles. Thirty-four studies were retrieved for a more detailed analysis; 36 studies were excluded due to a follow-up of less than five years, and another compared the mini-gastric bypass. In total, seven studies were included in the systematic review, but there was no significant difference in three of them.

**Conclusion:**

The gastric bypass had a greater weight loss than the vertical gastrectomy in all the evaluated studies.

## RESUMO

**Introdução:**

A cirurgia bariátrica é atualmente o tratamento padrão-ouro para o tratamento da obesidade não passível de controle clínico. As duas operações mais feitas são o bypass gástrico em Y-de-Roux e a gastrectomia vertical, e por isso tem sido foco de muita discussão.

**Objetivo:**

Comparar as duas técnicas em relação à perda de peso com pelo menos cinco anos de acompanhamento.

**Métodos:**

As buscas dos trabalhos foram realizadas nas bases de dados eletrônicas Medline, PubMed, Embase, SciElo, Lilacs, Cochrane de 2001 (início da gastrectomia vertical) até 2018, usando os seguintes descritores: “sleeve” ou “sleeve gastrectomy” combinada com “gastric bypass” ou “Roux-en-Y gastric bypass”,“weight loss” e “clinical trial”. Critérios de inclusão foram: trabalhos com pacientes com idade entre 18 e 65 anos; ensaio clínico; comparação entre as duas técnicas; acompanhamento mínimo de cinco anos; desfecho com avaliação de perda de peso.

**Resultados:**

A busca inicial identificou 1940 artigos, destes 185 publicações foram identificadas como ensaios clínicos. Cento e quarenta e um foram excluídos devido a que 67 não compararem as duas técnicas, 57 não abordaram a perda de peso e 17 por serem artigos repetidos. Foram recuperados 44 estudos para uma análise mais detalhada, 36 deles foram excluídos devido ao seguimento menor que cinco anos e outro por comparar o minibypass gástrico. No total sete estudos foram incluídos na revisão sistemática; no entanto, não houve diferença estatisticamente significativa em três deles.

**Conclusão:**

O bypass gástrico teve perda de peso maior que a gastrectomia vertical em todos os trabalhos avaliados.

## INTRODUCTION

Obesity is defined by excessive accumulation of potentially harmful body fat and classified by the World Health Organization as patients with body mass index (BMI) >30 kg/m^2 24^. The predictions are even worse: In 2025 the disease will affect one billion adults^23^. It is related as chronic systemic inflammation^14^ and metabolic disorders, among which the most common type 2 diabetes mellitus, hypertension and dyslipidemia^11^ ^,^ ^16^ ^,^ ^17^.

Clinical treatment is not effective for long-term sustained weight loss, as 95% of patients eventually regain their initial weight within two years^4^. Bariatric surgery has been considered the most effective method for treating long-term obesity, in improving the quality of life^15^, as well as in the remission of comorbidities that follow most cases^5^ ^,^ ^8^.

Currently the two most commonly performed bariatric surgeries in the world are Roux-en-Y gastric bypass (BGYR) and vertical gastrectomy (GV)^2^. Despite many controversies regarding the comparison of techniques, both are safe and effective, however with slightly different comorbidities remission rates^3^ ^,^ ^6^ ^,^ ^18^.

SG as a single procedure is a relatively new technique; was started in 2001 in the US and released in Brazil in 2010 by CFM^7^ ^,^ ^9^. It is undoubtedly the fastest growing operation in the world and has been the most performed in the USA since 2013^2^. However, there are many controversies mainly regarding the maintenance of long-term weight loss.

The recent introduction in Brazil - recent compared to the time of the other techniques - associated with the large increase in the number of procedures and adept surgeons, and the emergence of numerous controversies in the long-term weight loss, has led to the aim of this review.

## METHODS

The Medline, PubMed, Embase, SciElo, Lilacs, Cochrane electronic databases were retrospectively consulted from 2001 (beginning of SGS) to 2018 using the following descriptors: sleeve or sleeve gastrectomy combined with gastric bypass or “Roux-en-Y gastric bypass”, “weight loss” and “clinical trial”. Articles identified by the initial search strategy evaluated according to titles and abstracts, obeying the following inclusion criteria: 1) population aged 18 to 65 years; 2) articles with clinical trials; 3) surgical treatment comparing GV with BGYR; 4) patients with BMI greater than 35 kg/m^2^; 5) outcome with weight loss assessment. Exclusion criteria were: 1) animal studies; 2) non-surgical intervention (such as endoscopic gastroplasty) or other operations; 3) studies with follow-up of less than five years; 4) studies with designs other than clinical trials; 5) non-comparative studies between the two techniques. In cases where the title and abstract were not enlightening, we found necessary to read the full article. Study results were displayed by overweight loss (PEP%), BMI and weight

## RESULTS

### Study description

**FIGURE 1:**
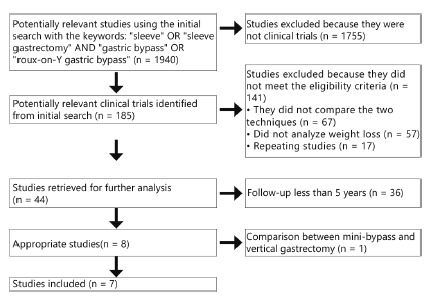
Flowchart of clinical trial selection

Figure 1 shows the flowchart of the initial search results to the selection of publications that were included for analysis and discussion. Initial research with the keywords “sleeve” OR “sleeve gastrectomy” AND “gastric bypass” OR “Roux-en-Y gastric bypass” identified 1940 articles. Seven studies selected at the end, published after 2010, and included 1014 patients in total, of whom 503 underwent BGYR and 511 GV. The sample size of the trials ranged from 64 to 240 patients. The more detailed characteristics of each study shown in Table 1.

**TABLE 1:** Characteristics of clinical trials comparing BGYR and GV

Reference	Year	N	BGRY	GV	RDZ	Country	Quality of life questionnaire	Complications	Follow-up 5º year	DM2 resolution or improvement	Statistic	Efficiency
Alexandrou et al ^1^	2017	180	73	107	NÃO	Greece (U)		BGYR > GV				BGYR > GV
Leyba et al ^13^	2014	117	75	42	NÃO	Venezuela (U)	-	BGYR = GV	63.2%	BGYR = GV	Wilcoxon test Fisher	BGYR = GV
Ignat et al ^10^	2016	100	45	55	SIM	France (U)	M-A-QoLQII GIQLI	BGYR>GV	-	-	T-student HLMs Bonferroni Wilcoxon test Fisher	BGYR > GV
Paterli et al ^19^	2017	217	110	107	SIM	Switzerland (M)	BARROS	BGYR>GV	34.5%	BGYR = GV	T-student Bonferroni Fisher’s	BGYR=GV
Salminen et al ^20^	2018	240	119	121	SIM	Finland (M)	M-A-QoLQII GIQLI		80.4%	BGYR = GV	Test - U ANOVA Kolmogorov-Smirnov test	BGYR=GV
Zhang et al ^25^	2014	64	32	32	SIM	China (U)	M-A-QoLQII	BGYR>GV	84.3%	BGRY>GV	T-student test-U Fisher	BGYR>GV
Schauer et al ^21^	2017	96	49	47	SIM	USA(U)	RAND 36-Item Health Survey		90%	BGYR>GV	Fisher SAS software	BGYR>GV
Total		1014	503	511								

BGYR=Roux-en-Y gastric bypass; GV=vertical gastrectomy; RDZ=randomized; (U) = single center; M=multicenter; Moorehead-Ardelt Quality of Life Questionnaire II (M-A-QoLQII) and Gastrointestinal Quality of Life Index (GIQLI)

Weight loss was assessed in most studies from the PEP% which is defined by: [(preoperative starting weight - current postoperative weight) / (starting weight - ideal weight)] x 100. Alexandrou et al.^1^ (2017) paper is a non-randomized clinical trial and has shown that BGYR is more efficient in long-term weight loss compared to GV (p<0.05). Zhang et al.^25^ (2014) and Ignat et al.^10^ (2016) from randomized controlled trials also observed higher PEP% in the BGYR group. Saminen et al.^20^ (2018) showed a greater tendency in weight loss with BGYR, however without statistically significant difference (p>0.05). The same result was identified in the non-randomized clinical trial of Leyba et al.^13^ (Table 2).

**TABLE 2:** Overweight loss% according to each clinical trial

	Year	BGYR	GV	p value	RDZ
Alexandrou et al.^1^	2017	78.4	55.8	<0.05	No
Zhang et al. ^25^	2014	76.2	63.2	<0.05	Yes
Ignat et al. ^10^	2016	74.8	65.1	0.045	Yes
Leyba et al. ^13^	2014	69.8	67.3	>0.05	No

Confidence interval of all studies=95%

Salminen et al.^20^ (2018), Paterli et al.^19^ (2017) and Schauer et al.^21^ (2017) are not shown in the table because they did not use the PEP% in their trials to report the difference in weight loss between the groups. Salminen et al.^20^ (2018) used the estimated average percentage of excess weight loss at five years. The average percentage was 57% (95% CI, 53-61%) after BGYR and 49% (95% CI, 45-52%) after SG. In five years, the estimate was 8.2 percentage units (95% CI, 3.2-1.2%) higher in the GBYR group than in the GV. However, the predefined clinical equivalence margins were -9 to +9 and based on these limits, the groups are not equivalent because the entire confidence interval is not within the margins. BGYR resulted in statistically greater weight loss than GV, but the difference was not significant. Peterli et al.^19^ (2017) assessed weight loss as excess percentage loss of BMI [initial BMI - current BMI] / (initial BMI - 25) x100]. In this study the excess loss of BMI for BGYR was 68.3% and for GV 61.1%, but without statistically significant difference (p=0.22). Schauer et al.^21^ (2017) in the STAMPEDE study compared weight loss from absolute weight. The initial mean weight and standard deviation of those submitted to BGYR was 106.8±14.9 and after five years decreased to 83.4±15.3 (difference of -2.2 in absolute weight, with deviation of ±9.6), whereas in GV the initial mean weight and the standard deviation were 100.4±16.8 and the weight after five years decreased to 81.9±15.0 (difference of -18.6 in the absolute weight and ±7.5 deviation) with p=0.01.

## DISCUSSION

Obesity is a chronic, serious, progressive disease that has no cure. Because of this, the increase in incidence in recent years has become a major public health challenge. Obesity patients undergoing surgical treatment have been treated more effectively and sustainably in the long term. However, when it comes to surgical treatment, there is a wide and variable range of possibilities around the world^2^ ^,^ ^12^. GV since its inception has been growing exponentially and together with BGYR make up the two most commonly performed techniques today, all around the world. However, despite the growth of GV many criticisms and controversies have been brought into discussion, especially regarding the maintenance of long-term weight loss after GV.

This systematic review sought clinical trials published in the world literature that compared techniques in relation to PEP% with follow-up for more than five years. In general, all seven selected studies show a trend of higher PEP% in patients undergoing BGYR, although three of these studies did not show statistically significant p^13^ ^,^ ^19^ ^,^ ^20^.

Leyba et al.^13^ (2014) performed the clinical trial with procedures performed by the same team, and the patients distributed according to their desire to perform BGYR or GV and after five years neither procedure was superior to the other in weight loss. However, they assume that there may have been selection bias in the distribution method, which eventually resulted in unequal size of the sample groups, BGYR with 75 and GV 42 patients.

The multicenter randomized large sample studies of Paterli et al.^19^ (2018) and Salminen et al.^20^ (2018) converge on results very similar to the study by Leyba et al.^13^ (2014). The results show equivalence between the two groups regarding the increase of quality of life, the number of reoperations or interventions and the decrease of the mean BMI value. Salminen et al.^20^ (2018), however, emphasize that the difference was not clinically significant due to predefined equivalence margins.

On the other hand, Alexandrou et al.^1^ (2017) after 180 consecutive procedures (73 BGYR and 107 GV), observed that patients with BMI between 35 and 55 had similar PEP% in both techniques in the first 12 months. However PEP% after BGYR was significantly higher than GV over the next four years. Thus, the study points out that the safety profiles of operations are similar, but BGYR achieved considerably higher PEP% when compared to long-term GV.

Ignat et al.^10^ (2016), Zhang et al. ^25^(2014), Schauer et al.^21^ (2017) are single center randomized controlled trials that demonstrated well-established results. In these studies, BGYR and GV are equally safe and effective in improving quality of life and resolving comorbidities. However, when analyzing the PEP%, the BGYR showed a statistically significant greater loss in five years.

The limitation of this review is that there is heterogeneity of the studies regarding the size of the samples, being different in operative techniques, surgeons, countries, centers, and in the way of measuring the variables. It is noted that three of these clinical trials failed to demonstrate statistically significant difference.

## CONCLUSION

There is a tendency for greater excess weight loss after five years of follow-up with Roux-en-Y gastric bypass in relation to vertical gastrectomy.
